# Objectively Measured Walking Duration and Sedentary Behaviour and Four-Year Mortality in Older People

**DOI:** 10.1371/journal.pone.0153779

**Published:** 2016-04-15

**Authors:** Jochen Klenk, Dhayana Dallmeier, Michael Dieter Denkinger, Kilian Rapp, Wolfgang Koenig, Dietrich Rothenbacher, ActiFE Study Group

**Affiliations:** 1 Institute of Epidemiology and Medical Biometry, Ulm University, Ulm, Germany; 2 Department of Geriatrics and Geriatric Rehabilitation, Robert-Bosch-Hospital, Stuttgart, Germany; 3 Department of Internal Medicine II-Cardiology, University of Ulm Medical Center, Ulm, Germany; 4 Agaplesion Bethesda Hospital, Geriatric Research Unit, Ulm University and Geriatric Center Ulm/Alb-Donau, Ulm, Germany; 5 Deutsches Herzzentrum München, Technische Universität München, Munich, Germany; 6 DZHK (German Centre for Cardiovascular Research), partner site Munich Heart Alliance, Munich, Germany; University of Rome Foro Italico, ITALY

## Abstract

**Background:**

Physical activity is an important component of health. Recommendations based on sensor measurements are sparse in older people. The aim of this study was to analyse the effect of objectively measured walking and sedentary duration on four-year mortality in community-dwelling older people.

**Methods:**

Between March 2009 and April 2010, physical activity of 1271 participants (≥65 years, 56.4% men) from Southern Germany was measured over one week using a thigh-worn uni-axial accelerometer (activPAL; PAL Technologies, Glasgow, Scotland). Mortality was assessed during a four-year follow-up. Cox-proportional-hazards models were used to estimate the associations between walking (including low to high intensity) and sedentary duration with mortality. Models were adjusted for age and sex, additional epidemiological variables, and selected biomarkers.

**Results:**

An inverse relationship between walking duration and mortality with a minimum risk for the 3rd quartile (102.2 to128.4 minutes walking daily) was found even after multivariate adjustment with HRs for quartiles 2 to 4 compared to quartile 1 of 0.45 (95%-CI: 0.26; 0.76), 0.18 (95%-CI: 0.08; 0.41), 0.39 (95%-CI: 0.19; 0.78), respectively. For sedentary duration an age- and sex-adjusted increased mortality risk was observed for the 4th quartile (daily sedentary duration ≥1137.2 min.) (HR 2.05, 95%-CI: 1.13; 3.73), which diminished, however, after full adjustment (HR 1.63, 95%-CI: 0.88; 3.02). Furthermore, our results suggest effect modification between walking and sedentary duration, such that in people with low walking duration a high sedentary duration was noted as an independent factor for increased mortality.

**Conclusions:**

In summary, walking duration was clearly associated with four-year overall mortality in community-dwelling older people.

## Background

Physical activity is an important component of health. The beneficial effects of a physically active lifestyle on various health outcomes have been investigated in many studies [1]. Strong evidence exists that physically active subjects have higher levels of physical fitness, better quality of life, and a lower risk for a variety of chronic diseases such as cardiovascular diseases, diabetes, cancer and others [2]. Furthermore, increased physical activity is closely linked to attenuating cognitive and functional decline over time [3], even in a dose-dependent fashion [4]. Physical activity has been discussed as an important step on the pathway to disability [5]. It has also been shown to be inversely related to inflammatory processes as well as mortality [6–9]. For older adults, the American College of Sports Medicine and the American Heart Association recommend moderate-intensity aerobic physical activity for a minimum of 30 min on five days each week or vigorous-intensity aerobic activity for a minimum of 20 min on three days each week [2].

However, data from older adults, which are at the highest risk for most of the diseases relevant in this context and in whom physical activity declines most [10], are still sparse. Importantly, most available large cohort studies including older adults have estimated physical activity using retrospective questionnaires [11,12]. This could cause information bias, particularly among older people, due to difficulties in exactly recalling everyday activities [13]. Only one study focusing on sedentary behaviour and mortality used accelerometry to assess physical activity [14]. Potential independent effects of physical activity and sedentary behaviour also represent controversial issues.

Further studies with prospective outcomes which might also provide specific insight in the underlying biologic mechanisms that translate the beneficial effects of physical activity into the health-related outcome such as mortality are needed. This knowledge can help to identify potential targets for intervention as well as help to evaluate the efficacy or effectiveness of measures. A first cross-sectional analysis of various markers in the ActiFE study indeed showed that walking duration as an important component of physical activity is associated with a more favourable profile of established and novel cardiovascular biomarkers in older persons [15]. However, data from a study with prospective outcomes would represent much stronger evidence.

Thus, the aim of this study was to analyse the effect of objectively measured walking duration (including low to high intensity) as well as sedentary duration on four-year mortality in a population of community-dwelling older people and also investigating the potential role of biomarkers and other functional measures representing different pathophysiologic pathways in this context.

## Methods

### Study population

The ActiFE Ulm (Activity and Function in the Elderly in Ulm) study is a population-based cohort study in older people aged 65 years or older, randomly selected in Ulm and adjacent regions in Southern Germany. Exclusion criteria were: being in residential care, severe deficits in cognition or serious German language difficulties. Between March 2009 and April 2010, 1,506 eligible individuals agreed to participate and underwent baseline assessments. The cohort and measurements taken have been previously described [16]. For the present analysis we excluded participants who did not complete the physical activity assessment (n = 173). Furthermore, since a previous analysis showed that physical activity on Sundays was considerably different to those on other days of the week, participants with less than two measurements on weekdays and one on a Sunday were excluded (n = 62). Overall, five or more complete days were available for 95% of the participants who completed the physical activity assessment. In total, 1271 participants were considered in the analyses (84.4% of total).

All participants provided written informed consent and the Ethics Committee of the University of Ulm had approved the study (application no. 318/08 and 50/12).

### Physical activity measurement

Physical activity was measured using a validated uni-axial accelerometer (activPAL, PAL Technologies Ltd., Glasgow, UK) [17]. The device was attached to the thigh using waterproof adhesive tape. Participants were instructed to wear the sensor over 24 hours for seven consecutive days. Only days with activity measurements over the full 24 hours were considered as a valid day and included in the analysis. Accordingly, the first and the last day of the assessment period were excluded. The data processing algorithm detects upright posture as well as walking patterns and classified the activity into three categories: (1) lying or sitting, (2) standing and (3) walking (including low to high intensity walking). Periods of lying or sitting were defined as sedentary periods. Average daily walking duration and average daily sedentary duration in minutes per day were calculated by weighting the available week days and the Sunday of each subject based on one week and used to quantify the individual’s physical activity level. Activity durations were estimated for the whole day (24 hours) as well as for the following clock time intervals of the day: 06:00-<24:00, 00:00-<06:00, 06:00-<12:00, 12:00-<18:00, 18:00-<24:00. For the analyses of each time interval average daily walking duration as well as average daily sedentary duration were categorized in time-interval-specific quartiles to be able to investigate non-linear associations.

### Covariates

Baseline assessments were completed by trained research assistants using standardised methods. Age, sex, socio-economic factors and co-morbidity were ascertained by self-report. Height and weight were measured to calculate body mass index (BMI). The mini-mental state examination (MMSE) was performed to assess cognitive status [18]. A threshold of <25 points was set to define cognitive impairment. Physical performance assessment included habitual walking speed as well as handgrip strength (JAMAR dynamometer, Sammons Preston, Bolingbrook, Illinois). Blood at baseline was drawn under standardized conditions, centrifuged, aliquoted and stored at -80°C. NT-proBNP was measured by electrochemoluminescence on an Elecsys 411 (Roche Diagnotics) (coefficient of variation (CV) < 5%, lower limit of detection (LOD) of 5 pg/ml). High-sensitive troponin I (hs-cTnI) was measured on an ARCHITECT STAT (Abbott Diagnostics), with a within-laboratory imprecision of ≤ 10% CV across the range of 10 to 50000 ng/L (LOD < 2.0 ng/L), and a reported within-run and within-laboratory CV < 5.5%. Hs-cTnT was also measured on an Elecsys 411 with a LOD equal to 5 ng/L. All other measurements were done by routine methods in a blinded fashion.

### Ascertainment of deaths

Mortality status and date of death were obtained from the local registration offices for all participants four years after the baseline measurement. Date of censoring was 14^th^ January 2014. Time to death or censoring was calculated for each participant.

### Statistical analysis

Characteristics of the study population were calculated for the total population as well as for each quartile of walking and sedentary duration including crude mortality rates per 1000 person-years. Median values and interquartile range for each quartile of walking and sedentary duration as well as Spearman rank correlation coefficients adjusted for age and sex were calculated for each included biomarker.

Cox-proportional hazards models were used to estimate the independent effect of both, walking and sedentary duration by quartiles, on all-cause four-year mortality. The first quartile served as reference for walking and sedentary, respectively. Different models were fitted separately for walking and sedentary duration: **Model 1** –adjusted for age and sex; **Model 2** –additionally to Model 1 adjusted for duration of school (≤9 yrs vs. other), smoking status (current smoker vs. other), alcohol intake (daily intake vs. other), BMI (per kg/m²), diabetes (yes, no), hypertension (yes, no), cardiovascular disease (yes (history of myocardial infarction or congestive heart failure or stroke), no), history of cancer (yes, no), or chronic kidney disease (yes, no); **Model 3** –additionally to Model 2 adjusted for selected biomarkers (all included biomarkers considered, details see S1 Table). Biomarkers were separately added to Model 2. Those which were associated with p<0.10 with the outcome were included to Model 3 (qualified biomarkers were finally log transformed NT-proBNP, hs cTnI, and blood glucose).

Furthermore, we evaluated the nonlinear association of walking and sedentary duration with mortality using cubic restricted splines with knots at the 5, 35, 65, and 95%, respectively adjusting initially for age and sex, and for all the variables considered in Model 2. Median values of walking and sedentary duration were used as references (HR 1.00). To reduce the effect of extreme values on the margins of the distribution we excluded 2.5% of the observations on each margin.

In a final model we evaluated potential effect modification by categorizing walking and sedentary duration in four groups: (1) walking quartile 1 AND sedentary quartile 1, (2) walking quartile 1 AND sedentary quartile 2–4, (3) walking quartile 2–4 AND sedentary quartile 1, (4) walking quartile 2–4 AND sedentary quartile 2–4.

We also examined the possible presence of reverse causation by excluding those with poor self-perceived health status at baseline as well as deaths within the first year of follow-up from the analysis. Collinearity between walking and sedentary duration was evaluated estimating the Spearman correlation coefficient as well as the variance inflation factor (vif). In addition, reverse causation due to an underlying disease might also be present with an associated decrease of physical activity at baseline as well as with an increased mortality. In this respect secondary analyses excluding persons with poor self-perceived health status at baseline and deaths during the first year of follow-up have been performed. All analyses were performed using SAS 9.2.

## Results

The study population consisted of 1271 subjects (717 men and 554 women, mean age = 75.6 (SD = 6.51) years) with complete data on physical activity and mortality. The distributions of characteristics stratified by quartiles of walking as well as sedentary duration are presented in Table 1. The average daily walking duration was 104.0 (SD = 40.3) minutes and the average daily sedentary duration was 1060.1 (SD = 109.5) minutes. Between 6:00 and 24:00 the proportions of time spend in sedentary and walking duration was 65.5% and 9.5%, respectively. Walking duration and sedentary duration were both associated with each other (Spearman correlation coefficient r = 0.65) but did not show a critical collinearity (vif = 2.16).

**Table 1:** Baseline characteristics by quartiles of walking duration and sedentary duration.

	Total	Quartile of walking duration [min]				Quartiles of sedentary (sitting/lying) duration [min]			
		1	2	3	4	1	2	3	4
		n = 317	n = 318	n = 318	n = 318	n = 317	n = 318	n = 318	n = 318
Characteristic	n = 1271	(3.7–76.1)	(76.1–102.2)	(102.2–128.4)	(128.4–290.5)	(711.1–984.0)	(984.0–1065.8)	(1065.8–1137.2)	(1137.2–1392.3)
Age (years), mean (SD)	75.6 (6.51)	79.2 (6.67)	75.7 (6.13)	74.8 (6.02)	72.6 (5.43)	74.1 (6.06)	75.0 (6.45)	75.7 (6.08)	77.4 (6.97)
Women, n (%)	554 (43.6)	135 (42.6)	147 (46.2)	139 (43.7)	133 (41.8)	194 (61.2)	146 (45.9)	128 (40.3)	86 (27.0)
Duration of school education ≤9 yrs, n (%)	718 (56.5)	180 (56.8)	177 (55.7)	175 (55.0)	186 (58.5)	199 (62.8)	183 (57.6)	163 (51.3)	173 (54.4)
Current smoker, n (%)	83 (6.5)	26 (8.2)	22 (6.9)	15 (4.7)	20 (6.3)	18 (5.7)	20 (6.3)	12 (3.8)	33 (10.4)
Daily alcohol consumption, n (%)	385 (30.3)	107 (33.8)	97 (30.5)	85 (26.7)	96 (30.2)	75 (23.7)	88 (27.7)	115 (36.2)	107 (33.7)
BMI (kg/m²), mean (SD)	27.6 (4.13)	29.2 (4.89)	27.9 (3.97)	27.3 (3.69)	26.2 (3.28)	26.3 (3.72)	27.1 (3.44)	28.1 (3.71)	29.0 (4.98)
≥30 kg/m². n (%)	308 (24.2)	122 (39.6)	88 (28.6)	57 (18.5)	41 (13.3)	47 (14.8)	62 (19.5)	82 (25.8)	117 (36.8)
Mini Mental State Examination <25, n (%)	59 (4.6)	21 (6.6)	14 (4.4)	8 (2.5)	16 (5.0)	15 (4.7)	14 (4.4)	17 (5.4)	13 (4.1)
Self-reported comorbidity, n (%)									
Hypertension	682 (53.7)	208 (65.6)	163 (51.3)	179 (56.3)	132 (41.5)	141 (44.5)	169 (53.1)	185 (58.2)	187 (58.8)
Cardiovascular disease	318 (25.0)	115 (36.3)	85 (26.7)	66 (20.8)	52 (16.4)	63 (19.8)	63 (19.8)	78 (24.5)	114 (35.9)
Cancer	232 (18.3)	71 (22.4)	58 (18.2)	62 (19.5)	41 (12.9)	47 (14.8)	58 (18.2)	59 (18.6)	68 (21.4)
Chronic kidney disease	43 (3.4)	20 (6.3)	14 (4.4)	4 (1.3)	5 (1.6)	3 (1.0)	8 (2.5)	10 (3.1)	22 (6.9)
Diabetes	179 (14.1)	73 (23.0)	54 (17.0)	31 (9.8)	21 (6.6)	32 (10.1)	19 (6.0)	60 (18.9)	68 (21.4)
Habitual walking speed [m/s], mean (SD)	0.98 (0.29)	0.83 (0.28)	0.99 (0.27)	1.01 (0.28)	1.06 (0.26)	1.00 (0.28)	1.02 (0.27)	0.98 (0.27)	0.90 (0.31)
Handgrip strength [kg], mean (SD)	32.2 (11.2)	30.2 (10.7)	31.7 (11.8)	33.4 (11.4)	33.5 (10.5)	29.4 (10.3)	32.7 (10.8)	32.7 (11.1)	34.1 (11.9)
Walking duration [min], mean (SD)	104.0 (40.3)	55.2 (16.7)	89.6 (7.6)	114.5 (7.5)	156.6 (25.4)	134.9 (38.2)	116.7 (32.8)	96.2 (27.2)	68.4 (28.3)
Sedentary duration [min], mean (SD)	1060.1 (109.5)	1159.6 (92.2)	1076.1 (86.6)	1032.7 (82.3)	972.5 (81.6)	918.4 (52.7)	1025.6 (23.2)	1098.1(21.3)	1198.1 (48.9)
Number of deaths, n	100	62	19	8	11	15	19	17	49
Follow-up duration [years], median (Q1-Q3)	4.00 (3.73–4.25)	3.82 (3.56–4.11)	4.00 (3.73–4.22)	4.03 (3.78–4.27)	4.11 (3.80–4.31)	4.03 (3.75–4.27)	4.03 (3.78–4.30)	3.99 (3.75–4.25)	3.90 (3.70–4.20)
Mortality rate per 1,000 person years (95% CI)	20.3 (16.5; 24.6)	54.7 (42.2; 69.6)	15.3 (9.2; 23.8)	6.3 (2.7; 12.4)	8.6 (4.3; 15.3)	11.9 (6.7; 19.6)	15.1 (9.1; 23.4)	13.7 (8.0; 21.9)	41.9 (31.1; 55.0)

In general, subjects in the 4^th^ quartile of walking duration as well as in the 1^st^ quartile of sedentary duration showed the most favourable cardio-metabolic risk factor and history of chronic diseases. BMI, comorbidity, habitual walking speed and hand grip strength showed a dose-response relationship with both exposure variables. During a median follow-up of four years 100 subjects died representing a mortality rate of 20.3 (95% confidence interval (CI): 16.5; 24.6) deaths per 1000 person-years. For walking duration we found the highest mortality rate among those in the first quartile, our reference group (54.7 (95% CI: 42.4; 69.6) deaths per 1000 person-years) and the lowest one in the third quartile (6.3 (95% CI: 2.7; 12.4) deaths per 1000 person-years). With respect to sedentary duration those in the first and fourth quartile had the lowest and highest mortality rate with 11.9 (95% CI: 6.7; 19.6) deaths per 1000 person-years and 41.9 (95% CI: 31.3; 55.0) deaths per 1000 person-years, respectively.

In S1 Table the association between selected biomarkers representing different pathophysiologic pathways were analysed. Age- and sex-adjusted correlation coefficients between walking duration and all biomarkers were statistically significant. In contrast, the associations of NT-proBNP, hs-cTnT, albumin-creatinine ratio (ACR), testosterone and FT3 with sedentary duration were not statistically significant.

Table 2 shows the hazard ratios (HR) of mortality according to quartiles of walking and sedentary duration for the total day (24 hours) and in the different clock time intervals. For walking duration over 24 hours an inverse association with risk of death was seen for all three models with the lowest mortality for the third quartile. The pattern was consistent but more pronounced when excluding the night time between 00:00 and <06:00. For Model 2 (fully adjusted model) the observed HRs for quartiles 2 to 4 compared to quartile 1 were 0.45 (95%-CI: 0.26; 0.76), 0.18 (95%-CI: 0.08; 0.41), 0.39 (95%-CI: 0.19; 0.78), respectively. When looking at 6-hour time intervals the afternoon walking duration (12:00-<18:00) showed statistical significant U-shaped associations for all models. The inclusion of the qualifying biomarkers (log NT-proBNP, hs cTnI, blood glucose) only slightly attenuated the association of walking duration with mortality. However, the overall patterns persisted.

**Table 2:** Multivariable analyses between walking and sedentary duration with all-cause mortality for 24 hours as well as stratified by specific periods with time-specific quartiles.

**Walking duration**				
**Model 1—adjusted for sex and age**	**Quartile 1**	**Quartile 2**	**Quartile 3**	**Quartile 4**
24 hours	1.00	**0.42 (0.25; 0.71)**	**0.18 (0.09; 0.39)**	**0.33 (0.17; 0.66)**
06:00-<24:00	1.00	**0.40 (0.24; 0.68)**	**0.16 (0.07; 0.35)**	**0.32 (0.16; 0.62)**
00:00-<06:00	1.00	0.70 (0.40; 1.24)	0.88 (0.48; 1.61)	1.14 (0.67; 1.94)
06:00-<12:00	1.00	**0.24 (0.13; 0.44)**	**0.41 (0.24; 0.69)**	**0.35 (0.19; 0.67)**
12:00-<18:00	1.00	**0.38 (0.22; 0.66)**	**0.20 (0.10; 0.41)**	**0.30 (0.15; 0.58)**
18:00-<24:00	1.00	**0.52 (0.32; 0.85)**	**0.47 (0.27; 0.84)**	**0.38 (0.20; 0.72)**
Model 2 –fully adjusted*	**Quartile 1**	**Quartile 2**	**Quartile 3**	**Quartile 4**
24 hours	1.00	**0.46 (0.27; 0.79)**	**0.21 (0.10; 0.45)**	**0.42 (0.21; 0.85)**
06:00-<24:00	1.00	**0.45 (0.26; 0.76)**	**0.18 (0.08; 0.41)**	**0.39 (0.19; 0.78)**
00:00-<06:00	1.00	0.66 (0.37; 1.19)	0.81 (0.43; 1.52)	1.13 (0.65; 1.94)
06:00-<12:00	1.00	**0.27 (0.14; 0.51)**	**0.50 (0.29; 0.85)**	**0.41 (0.21; 0.79)**
12:00-<18:00	1.00	**0.41 (0.23; 0.71)**	**0.24 (0.12; 0.49)**	**0.38 (0.19; 0.77)**
18:00-<24:00	1.00	**0.54 (0.32; 0.89)**	**0.55 (0.30; 0.99)**	**0.42 (0.22; 0.82)**
**Model 3 –fully adjusted and biomarkers**^#^	**Quartile 1**	**Quartile 2**	**Quartile 3**	**Quartile 4**
24 hours	1.00	0.58 (0.33; 1.02)	**0.30 (0.14; 0.66)**	**0.47 (0.23; 0.99)**
06:00-<24:00	1.00	**0.54 (0.31; 0.95)**	**0.26 (0.12; 0.60)**	**0.43 (0.21; 0.90)**
00:00-<06:00	1.00	0.96 (0.52; 1.78)	0.80 (0.42; 1.54)	1.31 (0.74; 2.34)
06:00-<12:00	1.00	**0.39 (0.20; 0.75)**	0.74 (0.42; 1.29)	0.51 (0.26; 1.03)
12:00-<18:00	1.00	**0.43 (0.25; 0.76)**	**0.33 (0.16; 0.70)**	**0.48 (0.23; 0.997**)
18:00-<24:00	1.00	0.64 (0.38; 1.08)	0.67 (0.37; 1.24)	**0.39 (0.19; 0.78)**
**Sedentary duration**				
**Model 1—adjusted for sex and age**	**Quartile 1**	**Quartile 2**	**Quartile 3**	**Quartile 4**
24 hours	1.00	1.02 (0.52; 2.01)	0.94 (0.47 (1.88)	**2.05 (1.13; 3.73)**
06:00-<24:00	1.00	1.19 (0.59; 2.37)	1.07 (0.53; 2.16)	**2.29 (1.25; 4.22)**
00:00-<06:00	1.00	0.68 (0.41; 1.11)	**0.51 (0.30; 0.88)**	0.50 (0.27; 0.90)
06:00-<12:00	1.00	0.87 (0.48; 1.58)	0.64 (0.34; 1.21)	1.50 (0.89; 2.55)
12:00-<18:00	1.00	0.71 (0.33; 1.55)	1.12 (0.57; 2.20)	**2.18 (1.19; 3.99)**
18:00-<24:00	1.00	1.04 (0.55; 1.94)	1.11 (0.62; 2.01)	1.40 (0.79; 2.46)
Model 2 –fully adjusted*	**Quartile 1**	**Quartile 2**	**Quartile 3**	**Quartile 4**
24 hours	1.00	0.96 (0.48; 1.92)	0.73 (0.36; 1.49)	1.63 (0.88; 3.02)
06:00-<24:00	1.00	1.07 (0.53; 2.16)	0.84 (0.41; 1.72)	1.79 (0.95; 3.37)
00:00-<06:00	1.00	0.62 (0.37; 1.04)	**0.46 (0.26; 0.80)**	**0.44 (0.24; 0.82)**
06:00-<12:00	1.00	0.80 (0.43; 1.48)	0.57 (0.30; 1.08)	1.30 (0.76; 2.25)
12:00-<18:00	1.00	0.69 (0.32; 1.50)	0.84 (0.42; 1.69)	1.68 (0.90; 3.16)
18:00-<24:00	1.00	1.08 (0.57; 2.06)	1.05 (0.57; 1.95)	1.20 (0.66; 2.21)
**Model 3 –fully adjusted and biomarkers**^#^	**Quartile 1**	**Quartile 2**	**Quartile 3**	**Quartile 4**
24 hours	1.00	0.98 (0.49; 1.98)	0.59 (0.28; 1.22)	1.52 (0.81; 2.83)
06:00-<24:00	1.00	1.10 (0.54; 2.24)	0.65 (0.31; 1.35)	1.62 (0.85; 3.07)
00:00-<06:00	1.00	0.62 (0.36; 1.06)	0.66 (0.37; 1.16)	**0.41 (0.22; 0.80)**
06:00-<12:00	1.00	0.68 (0.36; 1.28)	**0.44 (0.22; 0.88)**	1.16 (0.67; 2.02)
12:00-<18:00	1.00	0.69 (0.32; 1.51)	0.75 (0.36; 1.55)	1.49 (0.79; 2.81)
18:00-<24:00	1.00	1.09 (0.56; 2.11)	1.05 (0.55; 2.00)	1.37 (0.73; 2.56)

* Adjusted for age, sex, duration of school ≤9 years, smoking status, alcohol intake, BMI, diabetes, hypertension, cardiovascular disease (myocardial infarction, congestive heart failure, stroke), cancer, chronic kidney disease. N = 8 were excluded due to missing values of BMI.

^#^Adjusted for age, sex, duration of school ≤9 years, smoking status, alcohol intake, BMI, diabetes, hypertension, cardiovascular disease (myocardial infarction, congestive heart failure, stroke), cancer, chronic kidney disease, biomarkers p<0.10 (log transformed NT-proBNP, hs cTnI, blood glucose). N = 8 were excluded due to missing values of BMI.

Sensitivity analyses assessing potential reverse causation showed mostly a bias towards the null, meaning a slight reduction of the observed effects, for walking as well as for sedentary behaviour (see S2 Table).

For sedentary duration over 24 hours a statistically significantly increased HR was only observed for Model 1 in quartile 4 compared to quartile 1 (HR: 2.05 (95% CI: 1.13; 3.73)). After adjustments made in Model 2 and 3 the effect was attenuated and became non-significant with HR of 1.63 (95% CI: 0.88; 3.02) and 1.52 (95% CI: 0.81; 2.83), respectively. Also at different time intervals no consistent significant associations were found.

**Fig 1:**
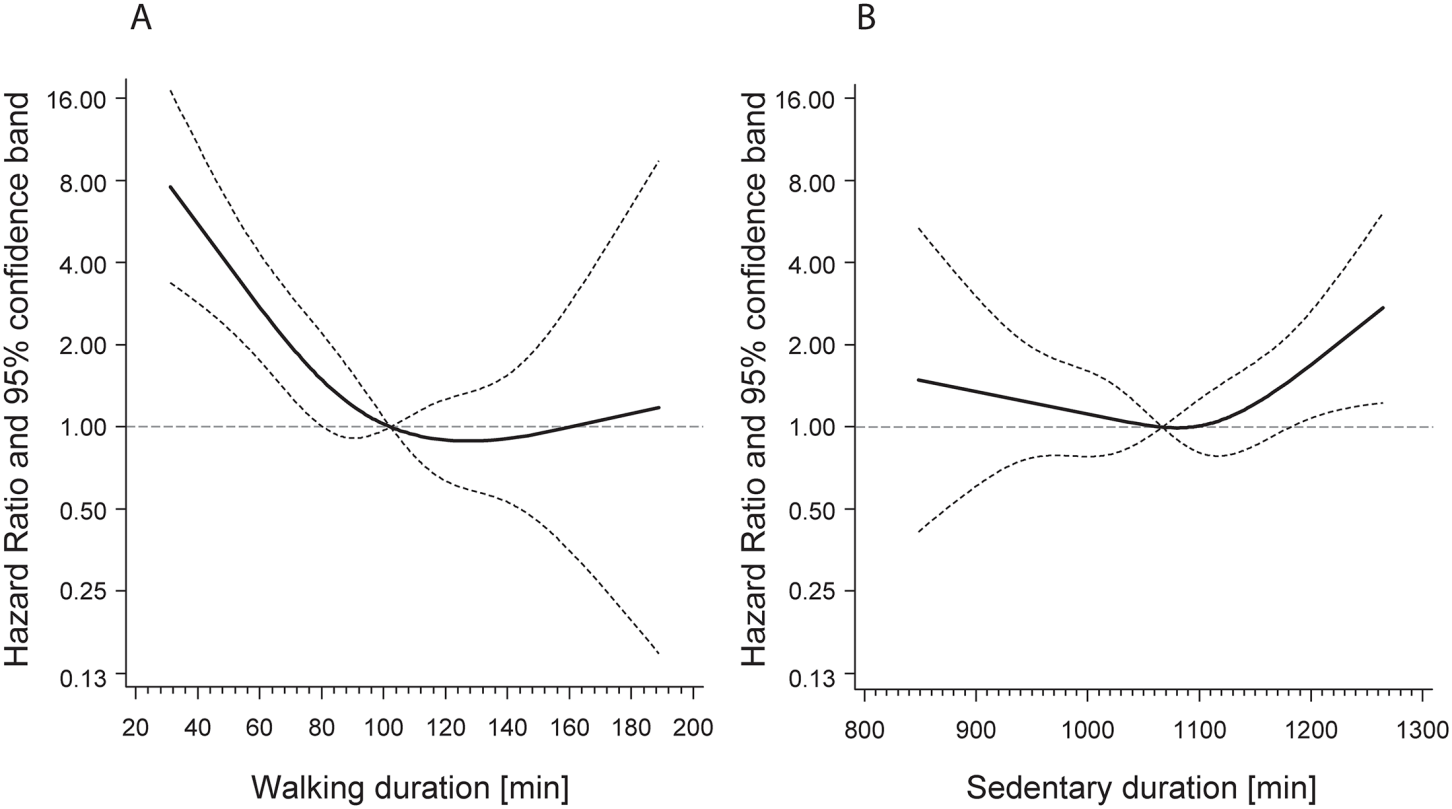
Association between walking duration (A) and sedentary duration (B) with mortality adjusted for age, sex, duration of school ≤9 years, smoking status, alcohol intake, BMI, diabetes, hypertension, cardiovascular disease (myocardial infarction, congestive heart failure, stroke), cancer, and chronic kidney disease. Walking and sedentary duration were modelled as continuous variables and fitted in Cox proportional hazard models using cubic restricted splines with knots at the 5, 35, 65, and 95%.

Fig 1 shows splines representing average daily walking duration (panel A) and average daily sedentary duration (panel B) with hazard for overall mortality after full adjustment (comparable to Model 2).

Table 3 summarizes the observed effects for walking and sedentary duration when considered both simultaneously in one model. In this analysis an increased mortality risk was only seen in the categories including the lowest quartile of walking duration with an increasing HR from 2.43 (95% CI: 1.32; 4.48) to 3.07 (95% CI: 1.85; 5.10) for increasing sedentary duration. The observed (3.07) and expected (1.10 * 2.43 = 2.67) joint effects are slightly different and may indicate the presence of possible effect modification.

**Table 3:** Joint association between average daily walking and sedentary duration with 4-year mortality fully adjusted model*.

	Sedentary duration*	
Walking duration*	Quartile 1–3	Quartile 4 (highest)
	Deaths/N	Deaths/N
	HR (95% CI)	HR (95% CI)
Quartile 2–4	32/807	6/109
	1.00 (Ref.)^#^	1.10 (0.46; 2.65)
Quartile 1 (lowest)	19/95	43/160
	2.43 (1.32; 4.48)	3.07 (1.85; 5.10)

^#^ Reference group

* Adjusted for age, sex, duration of school ≤9 years, smoking status, alcohol intake, BMI, diabetes, hypertension, cardiovascular disease (myocardial infarction, congestive heart failure, stroke), cancer, chronic kidney disease. N = 8 were excluded due to missing values of BMI.

## Discussion

In this large prospective cohort study involving 1,271 community-dwelling subjects aged 65 years and older living in Southern Germany we found an inverse relationship between objectively measured walking duration and four-year all-cause mortality. The minimum risk was observed for the 3^rd^ quartile, which corresponds to an average daily walking duration between 102.2 and 128.4 minutes. In contrast, for sedentary duration a significant increased mortality risk was observed only in the age-and sex adjusted model for the 4^th^ quartile (daily sedentary duration ≥1137.2 min.), which disappeared after full adjustment. Combining walking and sedentary duration according to extreme quartiles suggested the presence of effect modification such that an independent effect of sedentary time was especially evident in the group with lowest walking duration.

It is well accepted and has been consistently shown that physical activity is inversely associated with all-cause mortality [7]. However, there is still an ongoing discussion about recommended type, duration, and intensity of physical activity. Walking is the most frequently performed and one of the safest forms of physical activity [19]. There are only a few studies reporting an objective measurement of walking duration in older people [20]. Levels of average walking duration reported in our cohort are relatively high compared to populations of previous studies with the majority of people walking more than one hour per day [20]. However, our relatively healthy cohort consists of a broad range of activity levels, including also persons with low walking durations. Our results suggest that even low intensity activities such as walking are associated with a considerable reduction in mortality, which has widespread public health implications. Woodcock et al. observed the same pattern in a large meta-analysis including almost 1 million participants [8]. A study directly focusing on the effect of walking on mortality reported similar results [21]. While the meta-analyses included very broad age ranges, Landi et al. specifically followed older people aged 80 years or over for 24 months [19]. They found that walking at least 1h per day was associated with a relative risk of 0.36 (95% CI: 0.12; 0.98) compared to those walking less than 1h per day.

In contrast, the Copenhagen City Heart Study found a strong association between walking intensity and mortality but not with walking duration and mortality [22]. Results in men from the Harvard Alumni Health Study indicate that only moderate to vigorous activity is associated with a decrease in mortality [23]. However, the mean age of both cohorts was about 20 years lower compared to the ActiFE-cohort. Walking is a composition of low, moderate (brisk walking) and vigorous (running, brisk walking up a hill) physical activity. In our study, however, we were not able to differentiate between different levels of walking intensity. There is evidence, however, that the composition of activity changes with ageing, with a decline in physical activity mainly driven by reductions in moderate-to-vigorous activity [24].

The observed proportion of sedentary time as recorded in our study is in line with data from the US and Germany who reported that older adults spend about two thirds of the time awake in sedentary behaviour [25,26]. Our results regarding sedentary duration and mortality on the first glance seem in contrast to previous findings. Although we found an increased mortality risk for those with long sedentary durations, no dose-response association was present and the effect diminished after full adjustment. Several authors reported persistent positive dose-response relationships between sedentary time and mortality [14,27,28]. Two studies used objective data on sedentariness measured with an Actigraph sensor [14,27]. Koster et al. concluded that sedentary behaviour is a risk factor for mortality independent of moderate-to-vigorous physical activity [14]. However, the lack of a standard assessment procedure for assessing walking and sedentary behaviour with sensors limits the comparability of results between studies [29].

It is important to discuss whether sedentary behaviour and physical activity are directly related or if sedentary time yields complementary but different information than walking and both are required for a full assessment of physical activity [30]. Pavey et al. reported a significant interaction between walking duration, sitting time and mortality [31]. Our results support the observation that those who walk less and additionally have accumulated more sedentary bouts might have a higher risk of mortality. However, due to sample size limitations we were not able to deeper analyze this possible effect modification.

It is also very important to understand the pathological pathways of the observed associations. The patterns of walking duration in our cohort were partly affected by both, measures of glycemic control and of hemodynamic cardiac stress. The association was seen in a dose-response relationship and may explain partly the beneficial effects of physical activity described in the literature even in older adults. Sedentary behaviour is associated with high levels of body fat and blood glucose, increased prevalence of type 2 diabetes and cardiovascular outcomes [32]. Prolonged sitting was associated with inactivation of lipoprotein lipase and therefore with the subsequent deleterious impacts on lipid metabolism [33].

As suggested by many studies, including ours, walking might be a very promising intervention target, especially in older age groups and among sedentary individuals taking up physical activity [34]. Walking can be easily performed even in older age groups and does not have substantial contraindications and leads only rarely to adverse events like falls [35]. A review of 24 RCTs showed that walking interventions can improve aerobic capacity and reduce adiposity as well as diastolic blood pressure in sedentary individuals [36]. Duration of walking was also positively associated with a more beneficial profile of markers for haemostasis and inflammation in middle-aged men and women [37].

### Strengths & Limitations

Major strengths of this study are the objectively measured walking duration and a population register based mortality assessment after four years in a large and well-described population-based cohort. Especially in older adults sensor-based measurements can improve the assessment of physical activity considerably. In this group daily activity is mostly accumulated by regular everyday tasks like housework, shopping or visiting friends or family. These events are less memorable than structured activities such as attending exercise classes or playing sports [38]. Additionally, the definition of sedentary duration reported in the literature is mostly based on activity-count thresholds, which is known to be difficult and inconsistent [39]. The activPAL used in our study directly measures sedentary periods by detection of changes in thigh posture and not by using thresholds of activity counts like the ActiGraph device. Therefore, our approach minimizes exposure misclassification of ‘quite standing’ as sedentary behaviour.

The fact that physical activity was only measured during one week may be considered a limitation of the study. Longer assessment durations might have improved the results. However, a one-week physical activity measurement seems to be adequate to assess the average activity level of an individual and is in line with current recommendations [40]. Although accelerometry seems to be one of the most reasonable methods to quantify physical activity in observational studies [13], a decrease in detection-sensitivity was observed at slow walking speed [17]. This may have biased the results, as underlying disease could lead to a reduction in gait speed and thereby to an underestimation of walking in combination with an increased risk of death. In addition, we were not able to differentiate between different levels of walking intensity (low, moderate and high intensity). As there is evidence that walking intensity is related to all-cause mortality [34] the observed patterns for walking may change when considering level of intensity. Besides walking also other forms of physical activity (e.g. swimming, strength training) may also have beneficial effects on mortality. However, the activPAL sensor was not able to assess these activities. Furthermore, estimates of physical activity may vary between seasons due to a lower walking duration in winter [41]. However, measurements were equally distributed over the whole year. Regarding sedentary duration it was not possible to identify sleeping time. However, our sensitivity analysis excluding the time period between 0:00 and 06:00, which very likely includes most of the sleeping time, did not detect a change of the estimates.

Sensitivity analyses excluding those with poor self-perceived health status at baseline as well as deaths within the first year of follow-up showed a slight reduction of the observed effects. Therefore, reverse causation due to an underlying disease might partly explain the observed patterns. Further studies including more than one physical activity assessment as well as a longer follow-up duration are needed to address this issue. This would also increase the available number of events to sufficiently assessing possible effect modification between physical activity and sedentary behaviour or other factors such as age. Finally, the results of our study are limited to relatively healthy community-dwelling older people. The relationship between activity level and mortality may not be the same in other cohorts or in institutionalized older people.

## Conclusions

We found an association between walking duration and overall four-year mortality within community-dwelling older people. Increasing walking duration might therefore be a well-justifiable recommendation improving overall health in older people.

## Supporting Information

S1 Table Median (Q1-Q3) of biomarkers by quartiles of walking duration and sedentary duration as well as Spearman correlation coefficients adjusted for age and sex. (DOCX)

S2 Table Secondary multivariable analyses between walking and sedentary duration with all-cause mortality excluding those with poor self-perceived health status at baseline (n = 25, 6 deaths), as well as deaths within the first year of follow-up (n = 17). (DOCX)
