# Supplementary material for: Objectively Measured Walking Duration and Sedentary Behaviour and Four-Year Mortality in Older People
Source: PLoS One. 2016 Apr 15;11(4):e0153779. doi: 10.1371/journal.pone.0153779 (PMC4833405; doi:10.1371/journal.pone.0153779)
Supplement: S1 Table — (DOCX) [file pone.0153779.s001.docx]

S1 Table. Median (Q1-Q3) of biomarkers by quartiles of walking duration and sedentary duration as well as Spearman correlation coefficients adjusted for age and sex

|  | **Walking duration [min]** | | | | |
| --- | --- | --- | --- | --- | --- |
|  | **Quartiles** | | | | **r** |
|  | **1** | **2** | **3** | **4** |  |
|  | n = 317 | n = 316 | n = 316 | n = 316 |  |
| **Biomarker** | (0 - 76.1) | (76.1 - 102.2) | (102.2 - 128.4) | (≥128.4) | n=1271 |
| **Inflammatory markers** |  |  |  |  |  |
| CRP [mg/L] | 2.68 (1.13-5.48) | 1.80 (0.90-3.53) | 1.54 (0.82-3.06) | 1.27 (0.71-2.29) | -0.20 |
| WBC [Giga/L] | 6.80 (5.80-7.90) | 6.40 (5.60-7.40) | 6.30 (5.30-7.10) | 6.00 (5.20-6.90) | -0.17 |
| **Cardiac markers** |  |  |  |  |  |
| NT pro BNP [pg/ml] | 250.0 (111.5-680.5) | 161.0 (79.6-283.0) | 130.0 (79.9-246.0) | 119.5 (65.9-223.5) | -0.11 |
| hs Troponin T [ng/L] | 7.67 (2.50-16.15) | 2.50 (2.50-8.77) | 2.50 (2.50-8.03) | 2.50 (2.50-6.88) | -0.15 |
| hs Troponin I [ng/L] | 7.45 (5.00-12.50) | 5.90 (4.40-9.00) | 5.70 (4.40-8.00) | 5.40 (4.20-7.80) | -0.11 |
| **Lipids** |  |  |  |  |  |
| Cholesterol [mmol/L] | 5.10 (4.30-6.00) | 5.50 (4.70-6.20) | 5.40 (4.70-6.20) | 5.50 (4.80-6.20) | 0.08 |
| HDL cholesterol [mmol/L] | 1.30 (1.10-1.60) | 1.40 (1.20-1.70) | 1.50 (1.20-1.70) | 1.50 (1.30-1.80) | 0.18 |
| LDL cholesterol [mmol/L] | 3.20 (2.50-3.90) | 3.50 (2.80-4.10) | 3.40 (2.80-4.10) | 3.50 (2.90-4.10) | 0.07 |
| **Metabolic markers** |  |  |  |  |  |
| Glucose [mg/dl]^a^ | 100.0 (91.0-115.0) | 98.0 (89.0-110.0) | 96.0 (88.0-106.0) | 95.0 (88.0-102.0) | -0.16 |
| Uric acid [µmol/L] | 341.0 (284.0-401.0) | 321.5 (275.0-375.0) | 308.0 (262.0-376.0) | 310.0 (259.0-371.0) | -0.15 |
| **Kidney function** |  |  |  |  |  |
| Creatinine serum [µmol/L] | 96.0 (83.0-112.0) | 88.0 (77.0-101.0) | 83.0 (74.0-96.0) | 85.0 (75.0-96.0) | -0.22 |
| Cystatin C serum [mg/L] | 1.00 (0.86-1.18) | 0.88 (0.77-1.02) | 0.86 (0.75-0.96) | 0.81 (0.73-0.94) | -0.22 |
| ACR [mg/mmol] | 0.85 (0.49-2.01) | 0.69 (0.47-1.31) | 0.58 (0.41-1.12) | 0.61 (0.42-1.25) | -0.07 |
| **Endocrine markers** |  |  |  |  |  |
| Vitamin D [ng/ml] | 17.9 (13.1-22.3) | 19.2 (15.8-24.5) | 20.5 (16.1-25.1) | 23.0 (18.1-27.5) | 0.22 |
| PTH [pg/ml] | 38.5 (28.7-51.7) | 35.5 (28.7-44.4) | 34.9 (27.6-43.7) | 32.6 (26.1-39.5) | -0.14 |
| Testosteron [ng/ml]] | 2.03 (0.21-4.73) | 2.26 (0.22-5.09) | 2.71 (0.22-5.21) | 3.61 (0.23-5.69) | 0.10 |
| SHBG [nmol/L] | 58.7 (44.2-77.0) | 62.4 (45.2-80.4) | 60.2 (45.5-79.0) | 61.3 (46.9-82.3) | 0.12 |
| FT3 [pmol/L] | 4.45 (4.04-4.74) | 4.45 (4.13-4.88) | 4.56 (4.21-4.99) | 4.62 (4.30-4.93) | 0.09 |
| FT4 [pmol/L] | 17.4 (15.7-19.3) | 16.8 (15.2-19.0) | 16.7 (15.1-18.8) | 16.4 (14.8-18.5) | -0.10 |
|  | **Sedentariness (sitting/lying) duration [min]** | | | | |
|  | **Quartiles** | | | | **r** |
|  | **1** | **2** | **3** | **4** |  |
|  | n = 317 | n = 316 | n = 316 | n = 316 |  |
| **Biomarker** | (0 - 984.0) | (984.0 - 1065.8) | (1065.8 - 1137.2) | (≥1137.2) | n=1271 |
| **Inflammatory markers** |  |  |  |  |  |
| CRP [mg/L] | 1.37 (0.75-2.80) | 1.53 (0.80-3.08) | 1.71 (0.97-3.78) | 2.18 (1.08-5.08) | 0.17 |
| WBC [Giga/L] | 6.10 (5.20-7.20) | 6.20 (5.40-7.00) | 6.30 (5.70-7.40) | 6.60 (5.70-7.80) | 0.12 |
| **Cardiac markers** |  |  |  |  |  |
| NT pro BNP [pg/ml] | 140.5 (81.6-252.0) | 141.0 (82.0-291.0) | 146.5 (81.5-261.5) | 214.0 (86.8-578.0) | 0.03 |
| hs Troponin T [ng/L] | 2.50 (2.50-7.87) | 2.50 (2.50-8.33) | 2.50 (2.50-8.67) | 6.86 (0-15.5) | 0.05 |
| hs Troponin I [ng/L] | 5.40 (4.10-8.00) | 5.90 (4.30-8.10) | 5.90 (4.50-8.65) | 7.20 (4.90-12.20) | 0.09 |
| **Lipids** |  |  |  |  |  |
| Cholesterol [mmol/L] | 5.60 (4.80-6.30) | 5.60 (4.80-6.20) | 5.30 (4.50-6.10) | 5.00 (4.30-6.00) | -0.06 |
| HDL cholesterol [mmol/L] | 1.50 (1.30-1.80) | 1.50 (1.30-1.70) | 1.40 (1.20-1.60) | 1.30 (1.10-1.50) | -0.19 |
| LDL cholesterol [mmol/L] | 3.50 (2.90-4.10) | 3.50 (2.90-4.20) | 3.30 (2.70-4.00) | 3.20 (2.50-4.00) | -0.05 |
| **Metabolic markers** |  |  |  |  |  |
| Glucose [mg/dl]^a^ | 95.0 (87.0-104.0) | 95.0 (88.0-102.0) | 100.0 (89.5-114.5) | 100.0 (92.0-114.0) | 0.14 |
| Uric acid [µmol/L] | 293.0 (248.0-357.0) | 306.0 (264.0-365.0) | 324.5 (276.0-383.5) | 359.5 (307.0-420.5) | 0.19 |
| **Kidney function** |  |  |  |  |  |
| Creatinine serum [µmol/L] | 80.0 (71.0-93.0) | 84.0 (76.0-97.0) | 88.0 (78.0-103.0) | 96.0 (85.0-113.0) | 0.23 |
| Cystatin C serum [mg/L] | 0.83 (0.73-0.96) | 0.84 (0.75-0.98) | 0.88 (0.79-1.02) | 0.98 (0.82-1.15) | 0.17 |
| ACR [mg/mmol] | 0.67 (0.46-1.29) | 0.64 (0.42-1.26) | 0.67 (0.45-1.21) | 0.74 (0.44-1.82) | 0.01 |
| **Endocrine markers** |  |  |  |  |  |
| Vitamin D [ng/ml] | 21.4 (16.9-25.9) | 20.6 (16.4-25.5) | 20.2 (15.7-25.4) | 18.7 (14.1-23.8) | -0.14 |
| PTH [pg/ml] | 32.8 (26.1-40.8) | 34.6 (27.3-44.6) | 35.1 (28.8-43.8) | 37.5 (28.8-49.0) | 0.12 |
| Testosteron [ng/ml]] | 0.32 (0.16-4.65) | 2.83 (0.22-5.33) | 3.01 (0.23-5.21) | 3.74 (0.40-5.45) | -0.04 |
| SHBG [nmol/L] | 63.5 (47.6-87.0) | 63.3 (49.1-81.4) | 56.7 (43.9-77.8) | 57.8 (43.4-74.8) | -0.11 |
| FT3 [pmol/L] | 4.51 (4.16-4.88) | 4.55 (4.16-4.92) | 4.50 (4.18-4.88) | 4.51 (4.15-4.86) | -0.05 |
| FT4 [pmol/L] | 16.7 (14.8-18.7) | 16.9 (15.3-18.8) | 16.7 (15.2-18.8) | 17.2 (15.3-19.1) | 0.08 |

^a^ Only participants with fasting blood glucose (last food intake ≥8 hours) were considered (n=826)
