# Supplementary material for: Objectively Measured Walking Duration and Sedentary Behaviour and Four-Year Mortality in Older People
Source: PLoS One. 2016 Apr 15;11(4):e0153779. doi: 10.1371/journal.pone.0153779 (PMC4833405; doi:10.1371/journal.pone.0153779)
Supplement: S2 Table — (DOCX) [file pone.0153779.s002.docx]

S2 Table. Secondary multivariable analyses between walking and sedentary duration with all-cause mortality excluding those with poor self-perceived health status at baseline (n=25, 6 deaths), as well as deaths within the first year of follow-up (n=17).

| **Walking duration** | | | | |
| --- | --- | --- | --- | --- |
| **Model 1 - adjusted for sex and age** | **Quartile 1** | **Quartile 2** | **Quartile 3** | **Quartile 4** |
| Full sample | 1.00 | **0.42 (0.25; 0.71)** | **0.18 (0.09; 0.39)** | **0.33 (0.17; 0.66)** |
| Exclude poor self-perceived health status | 1.00 | **0.48 (0.28; 0.82)** | **0.22 (0.10; 0.46)** | **0.39 (0.20; 0.77)** |
| Exclude 1 year deaths | 1.00 | **0.44 (0.25; 0.78)** | **0.22 (0.10; 0.47)** | **0.40 (0.20; 0.79)** |
| **Model 2 – fully adjusted*** | **Quartile 1** | **Quartile 2** | **Quartile 3** | **Quartile 4** |
| Full sample | 1.00 | **0.46 (0.27; 0.79)** | **0.21 (0.10; 0.45)** | **0.42 (0.21; 0.85)** |
| Exclude poor self-perceived health status | 1.00 | **0.54 (0.31; 0.94)** | **0.25 (0.12; 0.53)** | 0.53 (0.25; 1.06) |
| Exclude 1 year deaths | 1.00 | **0.46 (0.25; 0.83)** | **0.23 (0.11; 0.50)** | **0.44 (0.21; 0.92)** |
| **Model 3 – fully adjusted and biomarkers^#^** | **Quartile 1** | **Quartile 2** | **Quartile 3** | **Quartile 4** |
| Full sample | 1.00 | 0.58 (0.33; 1.02) | **0.30 (0.14; 0.66)** | **0.47 (0.23; 0.99)** |
| Exclude poor self-perceived health status | 1.00 | 0.70 (0.39; 1.24) | **0.36 (0.16; 0.79)** | 0.59 (0.28; 1.26) |
| Exclude 1 year deaths | 1.00 | 0.57 (0.31; 1.05) | **0.32 (0.14; 0.70)** | 0.47 (0.22; 1.01) |
| **Sedentary duration** | | | | |
| **Model 1 - adjusted for sex and age** | **Quartile 1** | **Quartile 2** | **Quartile 3** | **Quartile 4** |
| Full sample | 1.00 | 1.02 (0.52; 2.01) | 0.94 (0.47 (1.88) | **2.05 (1.13; 3.73)** |
| Exclude poor self-perceived health status | 1.00 | 0.94 (0.47; 1.89) | 0.93 (0.47; 1.87) | 1.76 (0.96; 3.23) |
| Exclude 1 year deaths | 1.00 | 0.96 (0.48; 1.90) | 0.65 (0.31; 1.40) | 1.63 (0.88; 3.00) |
| **Model 2 – fully adjusted*** | **Quartile 1** | **Quartile 2** | **Quartile 3** | **Quartile 4** |
| Full sample | 1.00 | 0.96 (0.48; 1.92) | 0.73 (0.36; 1.49) | 1.63 (0.88; 3.02) |
| Exclude poor self-perceived health status | 1.00 | 0.90 (0.45; 1.80) | 0.72 (0.35; 1.47) | 1.36 (0.72; 2.56) |
| Exclude 1 year deaths | 1.00 | 0.90 (0.45; 1.82) | 0.55 (0.25; 1.19) | 1.42 (0.75; 2.68) |
| **Model 3 – fully adjusted and biomarkers^#^** | **Quartile 1** | **Quartile 2** | **Quartile 3** | **Quartile 4** |
| Full sample | 1.00 | 0.98 (0.49; 1.98) | 0.59 (0.28; 1.22) | 1.52 (0.81; 2.83) |
| Exclude poor self-perceived health status | 1.00 | 0.90 (0.44; 1.85) | 0.56 (0.27; 1.18) | 1.26 (0.66; 2.39) |
| Exclude 1 year deaths | 1.00 | 0.94 (0.46; 1.92) | 0.46 (0.21; 1.03) | 1.41 (0.74; 2.68) |

^*^ Adjusted for age, sex, duration of school ≤9 years, smoking status, alcohol intake, BMI, diabetes, hypertension, cardiovascular disease (myocardial infarction, congestive heart failure, stroke), cancer, chronic kidney disease. N=8 were excluded due to missing values of BMI.

^#^ Adjusted for age, sex, duration of school ≤9 years, smoking status, alcohol intake, BMI, diabetes, hypertension, cardiovascular disease (myocardial infarction, congestive heart failure, stroke), cancer, chronic kidney disease, biomarkers p<0.10 (log NT-proBNP, hs cTnI, blood glucose). N=8 were excluded due to missing values of BMI.
